# Diagnostic System for Early In Situ Melanoma Detection Using Acoustic Microscopy and Infrared Spectroscopic Mapping Imaging

**DOI:** 10.3390/cancers17152599

**Published:** 2025-08-07

**Authors:** Georgios th Karagiannis, Ioannis Grivas, Anastasia Tsingotjidou, Georgios Apostolidis, Eirini Tsardaka, Ioanna Dori, Kyriaki-Nefeli Poulatsidou, Ioannis Tsougos, Stefan Wesarg, Argyrios Doumas, Panagiotis Georgoulias

**Affiliations:** 1 “ORMYLIA” Foundation, 63071 Ormylia, Greece; gapostolidis@diagnosismultisystems.eu (G.A.); e.tsardaka@artdiagnosis.gr (E.T.); 2 Diagnosis Multisystems, 63200 Nea Moudania, Greece; 3 Laboratory of Anatomy, Histology and Embryology, School of Veterinary Medicine, Faculty of Health Sciences, Aristotle University of Thessaloniki, 54124 Thessaloniki, Greece; janos@vet.auth.gr (I.G.); astsing@vet.auth.gr (A.T.);; 4 Laboratory of Experimental Neurology and Neuroimmunology, Medical School, Aristotle University of Thessaloniki, 54124 Thessaloniki, Greece; kpoulats@auth.gr; 5 Medical School, University of Thessaly, 41334 Larissa, Greece; tsougos@uth.gr (I.T.); pgeorgoul@med.uth.gr (P.G.); 6 Competence Center Visual Healthcare Technologies of Fraunhofer IGD, 64283 Darmstadt, Germany; stefan.wesarg@igd.fraunhofer.de; 7 Radiology-Medical Physics and Informatics Sector, Medical School, Aristotle University of Thessaloniki, 54124 Thessaloniki, Greece; drdumas@the.forthnet.gr

**Keywords:** acoustic microscopy, infrared spectroscopy, spectroscopic mapping imaging, microtomography, melanoma early detection, noninvasive examination

## Abstract

This study proposes a novel diagnostic system for the early detection of cutaneous melanoma based on morphological and biochemical changes during tumor formation. The methods used in this system are acoustic microscopy and infrared (IR) spectroscopy. The former identifies the anatomical parameters of the developing tumor, whilst the latter identifies its biochemical features, both at the micron scale. To implement this diagnostic method, an animal model that mimics human melanoma was developed. The results of this investigation show that using high-frequency (>20 MHz) acoustic microscopy in conjunction with spectroscopic images provides useful information about distinct features of melanoma tumors’ 3D structures. The structures and cytoarchitecture of the tumors were assessed using conventional histology, and their malignant nature was confirmed using histological and immumohistochemical analysis. The proposed approach may provide an invaluable tool in diagnostic dermatology, as it is noninvasive and produces highly detailed and accurate data about the early appearance and development of melanoma tumors.

## Simple Summary

This study presents a new noninvasive method for early detection of skin cancer (melanoma), combining acoustic microscopy and infrared spectroscopy. Acoustic microscopy uses sound waves to capture detailed images of the tumor’s structure, while infrared spectroscopy analyzes chemical changes. Researchers tested this technique on animal models resembling human melanoma. The findings show that high-frequency acoustic imaging (>20 MHz) combined with spectroscopy provides clear, accurate, and detailed three-dimensional images of tumors at early stages. Histological tests confirmed the cancerous nature of the tumors, proving this approach could significantly improve early melanoma diagnosis in dermatology.

## 1. Introduction

Skin cancer is one of the most common types of human malignancies worldwide, according to WHO and Skin Cancer Foundation statistics. Although melanoma represents only 1% of skin cancer cases, it is the leading cause of skin cancer deaths [1]. It is among the few types of cancer whose incidence has increased significantly in recent decades and continues to grow, though at a slower pace, mostly in women more than 50 years old [1,2,3,4,5,6,7,8,9]. It involves the transformation of epidermal melanocytes into melanoma cells. A neoplastic tumor grows in the superficial skin layers, infiltrates the deep layers and rapidly metastasizes to distant body sites. Melanoma at the local stage, which is confined to the epidermis, is considered curable, whereas once it has metastasized, effective treatment is more difficult. The 5-year relative survival rate for melanoma decreases from >99% for cases diagnosed at a localized stage to 35% for distant-stage disease. Therefore, early diagnosis is essential for a positive prognosis with this disease. Currently, a diagnosis is made by dermatologists on the basis of morphological criteria, followed by a biopsy [10]. Researchers have recently focused on the application of noninvasive imaging techniques for cutaneous melanoma diagnosis in an effort to reduce the number of routine skin biopsies and enhance the accuracy and reliability of early melanoma detection [11].

From the early 1990s until the mid-2000s, acoustic microscopy was used for the detection of melanoma [12,13,14,15,16,17]. Since 2000, this has been supplemented by photoacoustic techniques, mainly in a research context [18,19,20,21]. These techniques provide mainly tomographic information. Recently, significant research efforts have improved the technological aspects of melanoma diagnosis using a combination of ultrasonics, photoacoustics and Optical Coherence Tomography techniques [22,23,24,25,26,27,28,29,30,31,32]. Although the light absorption provides some information about the chemical composition of tissue [33,34,35], more detailed biochemical data can be acquired using the more efficient Fourier transform infrared spectroscopy (FT-IR), which was chosen for use in this study. Recent research using spectroscopic imaging techniques has aimed to provide qualitative information on the physicochemical and physiological characteristics of malignant tumors ([36,37,38,39,40,41,42,43] and more recently [44,45,46,47,48]). Besides FT-IR spectroscopy, there are also efforts to improve the detection of skin cancer using other spectroscopies like Raman spectroscopy, with one of the most recent reviews presented in [49]. However, the proposed method involves FT-IR spectroscopy mainly due to its potential to identify organic materials.

The capability to differentiate benign skin lesions from malignant melanoma, mainly using the high-frequency ultrasonic modality, is documented in previous research [26,50,51,52,53,54].

This study focuses on the combined use of two modalities, acoustic microscopy and infrared (IR) spectroscopy, for the in vivo assessment of changes in cutaneous melanomas’ morphological and biochemical features at the very early stages of tumor formation, providing a new approach to determining the examined nevi’s malignancy. Thus, compared to the most recent existing method, photoacoustic imaging using an infrared spectrophotometer, the proposed method is in a better position to identify chemical differentiations between the tumor and the healthy skin. Previous studies have found that light in the infrared and mid-infrared areas of the spectrum penetrates skin to at least the depth under investigation in this study [55,56,57,58,59,60,61,62,63,64,65,66,67,68,69].

At the same time, ultrasonics provide a clear and high-resolution depth profile of a structure. In order to efficiently test the proposed method, an animal model was developed to closely mimic this human malignancy; therefore, the data provided by this study enable us to evaluate the method’s potential for use in human dermatology. The paper is structured as follows: In Section 2, the diagnostic system is described. In Section 3, the animal model and the modalities applied in early melanoma diagnosis are presented and evaluated. Following this, in Section 3.3 the presented system is evaluated by applying it to melanoma cases in mice. In Section 4, issues concerning the methods and devices used are discussed. Finally, in Section 5 the conclusions are presented.

## 2. Diagnostic System

**Figure 1:**
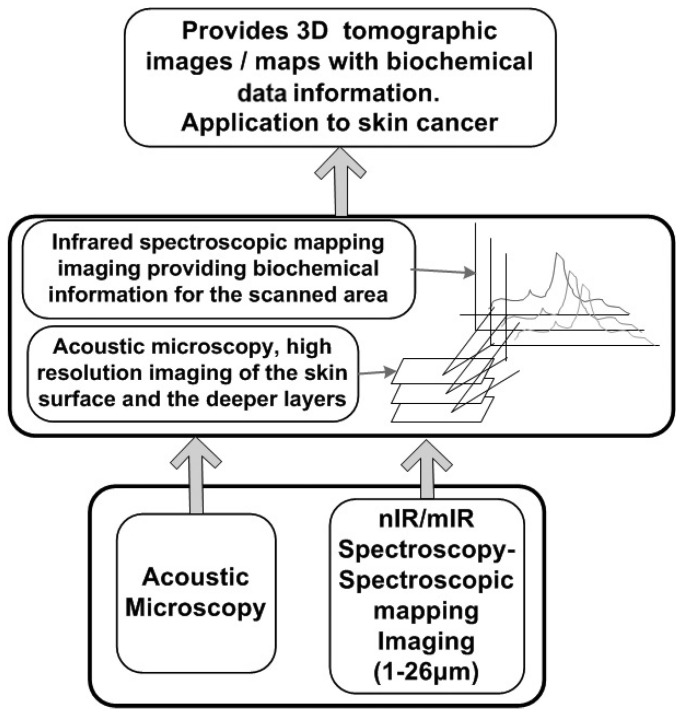
Overview of the diagnostic concept.

The proposed system for melanoma detection consists of two basic modules: acoustic microscopy and infrared spectroscopy in the form of spectroscopic mapping imaging. Using acoustic microscopy, tomographic information from the skin is acquired, while using infrared spectroscopic mapping imaging in the diffuse reflectance or reflectance mode, biochemical alterations in the skin during tumor formation are detected. In addition, the data from both modules are processed using the appropriate methods to reveal the information needed to diagnose malignancy. An overview of this method for the early detection of melanoma and its constituents is shown in Figure 1.

### 2.1. Acoustic Microscopy

**Figure 2:**
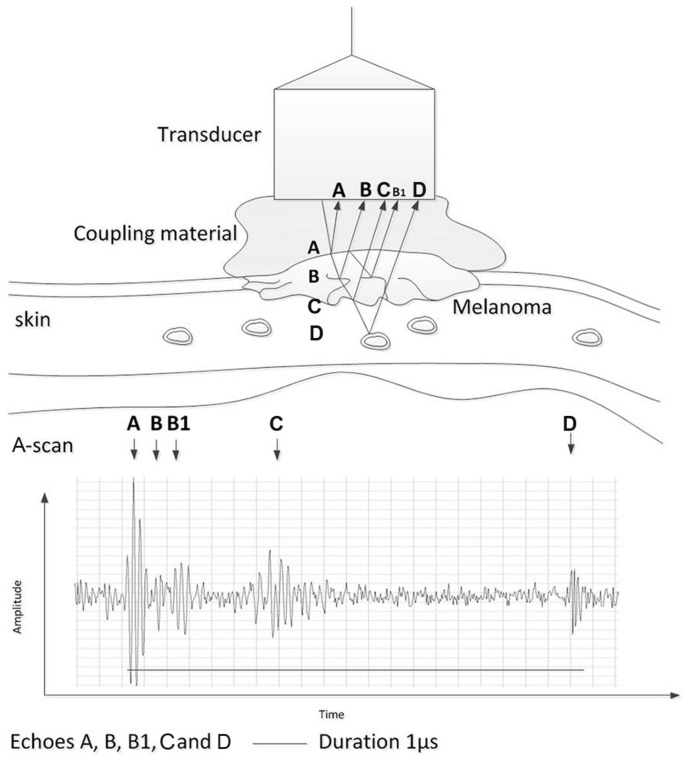
Propagation of acoustic wave through skin layers and production of A-scans from reflected components of initial wave.

In the simplest and most widely employed non-destructive testing (NDT) applications, a single piezoelectric transducer is used to excite an acoustic wave into the object under investigation. In many cases, the transducer is placed inside a water bath, where the wave propagates through the water medium into the object [70,71]. Generally, it is crucial that the acoustic impedances in the contact between the transducer and the object investigated, in this case a skin tumor, match for the optimum propagation of the ultrasonic waves from the transducer to the object, especially in high-frequency ultrasonics. In this case, hydrogel was used for the coupling of the tissue—skin—and the transducer. Supposing that the acoustic source is excited by a short electrical pulse, the wave generated by the transducer will be a short acoustic pulse, which will propagate through the skin melanoma [72] and eventually be reflected and scattered due to acoustic impedance discontinuities between the differently sized internal structures of the skin melanoma (Figure 2).

The reflected signal is named an A-scan and is a single, time-resolved signal plotting the amplitude as a function of the depth reached at a single location. The time difference of an echo (time-of-flight (TOF)) in the recorded signal can be used to determine the distance of the flaw (from the transducer). A B-mode scan is a series of A-scans along a single line. Usually, it is generated by plotting log-compressed images as a function of the depth and lateral position. Finally, a C-scan is a series of B-scans. To form a 2D image, usually the maximum amplitude or the amplitude at a certain depth is plotted. A C-scan can also form 3D images, which were the type of images used in this study.

### 2.2. Infrared Spectroscopic Mapping Imaging

**Figure 3:**
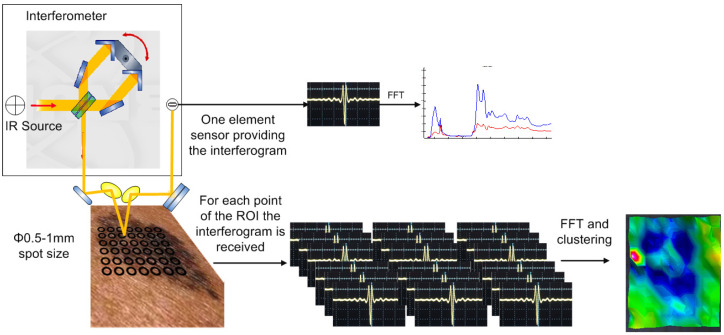
Principle of infrared (IR) spectroscopic mapping imaging. The orange line is the optical-beam path and the red line indicates the direction of the illumination from the source to the interferometer.

An IR spectrometer is composed of three basic optical elements: (a) an IR source; (b) an interferometer, which comprises a beam splitter and two mirrors, one fixed and one moving; and (c) a detector. The working principle of an IR spectrometer is illustrated in Figure 3. The beam produced by the IR source passes through an aperture. Then, it is optically filtered before entering the interferometer, which constitutes the heart of the spectrometer. The motion of the interferometer’s moving mirror modulates the light beam, which then exits the interferometer and is led to the tumor along an optical path. The light is reflected by the tumor and finally guided to the detector, which measures its intensity, i.e., its power. The signal at the detector, i.e., the interferogram, is amplified, filtered and digitized before being processed to produce the tumor’s spectrum. The selection of the three optical elements’ technical characteristics depends on the spectral region, i.e., near-IR (nIR), mid-IR (mIR) or far-IR (fIR), to be measured. The interferometer’s main working principles are the same as those of the two-beam interferometer originally designed by Michelson in 1891, which its design is based on [73].

**Figure 4:**
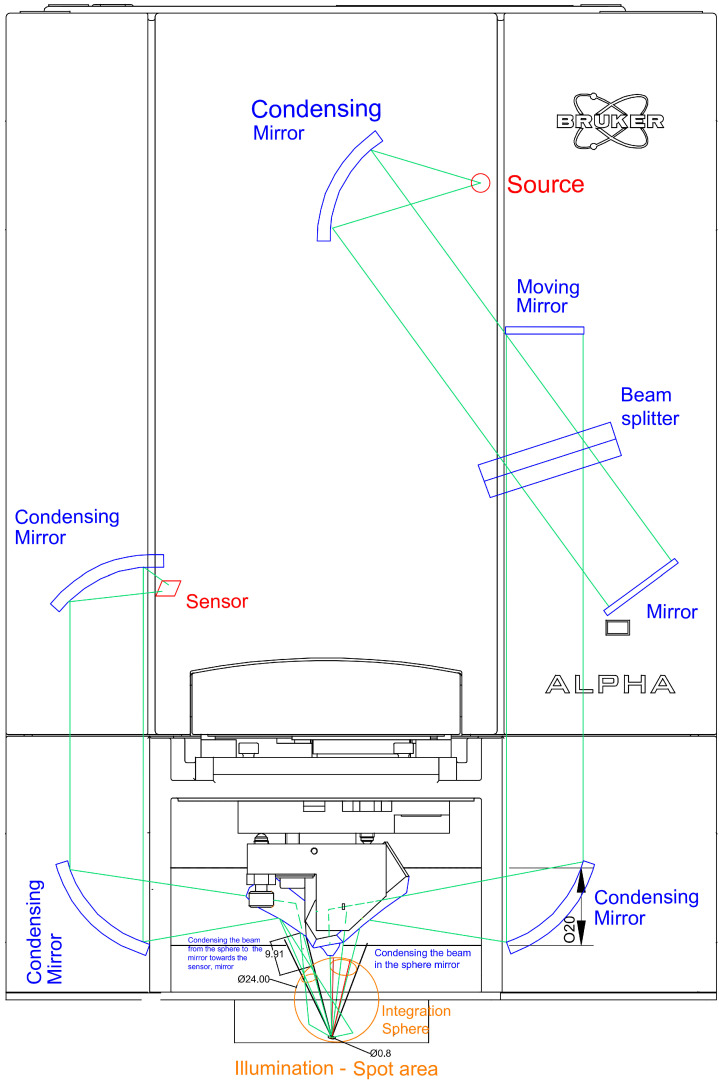
Top view of the adapted infrared (IR) spectrophotometer with an integration sphere. The optical beam path is shown in green, the integration sphere in orange, the optical components in blue and the source and sensor in red.

**Figure 5:**
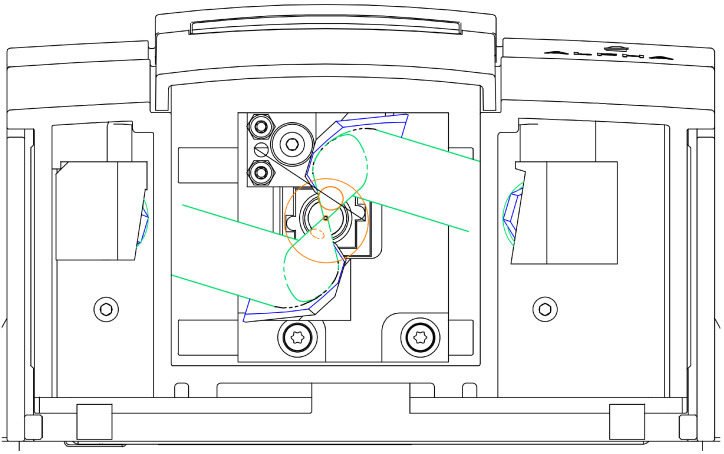
View of the sampling area of the adapted infrared (IR) spectrophotometer with an integration sphere. The optical beam path is shown in green, the integration sphere in orange and the optical components in blue.

**Figure 6:**
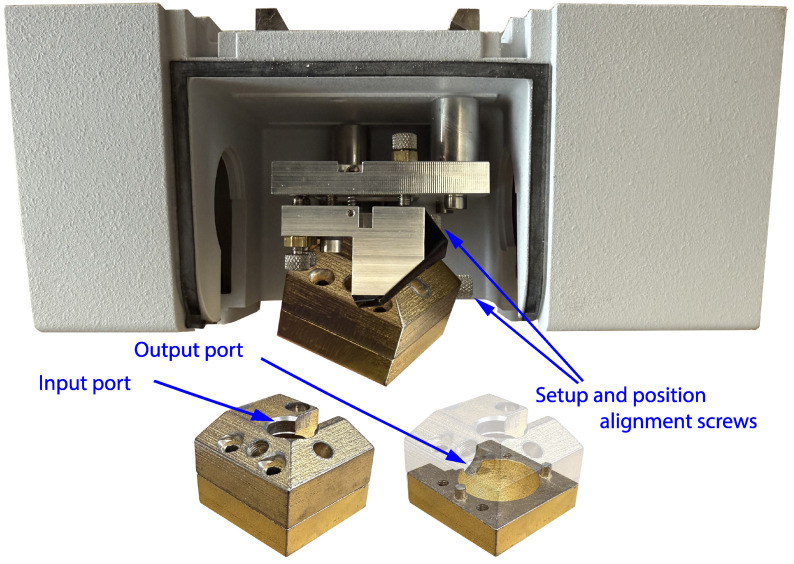
Reflectance module of adapted infrared (IR) spectrophotometer with integration sphere.

The system is based on a Bruker ALPHA spectrophotometer equipped with a reflectance module. To measure the diffuse reflectance of the sample from the final beam, guided from the emitting gold-coated mirror to the sample to be reflected back to the collecting gold-coated mirror, an integrating sphere is set up with a diameter of 24 mm. It is based on an old integration sphere that was used in the laboratory in conjunction with Bruker spectrometers [74,75]. A diagram of the exact design is provided in Figure 4 and Figure 5. The emitted light is guided into the integration sphere, the focal length and spot area of which are designed to be the same as those of the original one in the ALPHA spectrophotometer. Using the integration sphere, all the angles of the light reflected from the sample are integrated, minimizing the effects of the specular reflectance and therefore increasing the repeatability and reliability of the measurement. When using the integration sphere, the specular reflectance is not guided into the output port, and the sphere outputs a small part of the reflectance, reduced dramatically after many reflections inside it, if not totally omitted. Then, the diffuse reflectance from the sphere is guided through the output port to the ALPHA condensing receiving mirror, which guides it to the sensor via the conventional optical path. The output port is 8 mm, and the distance between the condensing mirror and the sensor is equal to or less than 9.9 mm, ensuring the collection of the diffused reflectance flux from the mirror. The red lines in the figures show the specular reflectance, and the orange lines show beams of light that are guided to the sample and the sensor in the way described above. A probe based on the aforementioned design is shown in Figure 6.

### 2.3. Data Processing

The data acquired from both modules are processed accordingly to obtain information from the structure of the healthy and diseased skin. The three-dimensional morphology of the measured skin region is reconstructed based on data acquired using the acoustic microscope, following a preprocessing phase. During preprocessing, background distortion that occurs due to the housing of the transducer is subtracted from the measurement. Although this distortion seems constant, it is affected by the conditions under which a specific measurement is taken, e.g., the temperature. As a result, it is estimated by exciting the coupling material without the object present but under the same conditions and averaging the measurements. Also, high-frequency noise is suppressed using low-pass filtering, with the cut-off frequency dependent on the specifications of the transducers. On the other hand, spectroscopic data, i.e., the acquired spectra, from the measured skin regions are subjected to a clustering procedure to identify particular points where biochemical changes occur. In particular, the clustering procedure used is a fuzzy C-mean (FCM) clustering algorithm [74]: assuming that $I=N_{x}\times N_{y}$ spectrum measurements are taken within the ROI of a melanoma site, the FCM algorithm takes *I* vectors, $y_{i}\left[n\right]$, with *N* dimensions and finds *L* clusters with the centroids $k_{l}\left[n\right]$. It also finds the degree of membership, $F_{i,l}$, of each vector, $y_{i}\left[n\right]$, to the $l^{th}$ cluster, where $F_{i,l}\in \left(0,1\right)$ and $\sum_{l}F_{i,l}=1$. The FCM algorithm minimizes the following objective function:

(1) $$J_{FCM}=\sum_{i=1}^{I}\sum_{l=1}^{L}F_{i,l}^{m}\cdot d_{i,l}^{2},$$

where *m* is a predefined fuzzifier, chosen to be equal to 2. Also, $d_{i,l}^{2}$ is the Euclidean distance from the $i^{th}$ vector to the $l^{th}$ cluster centroid. The steps in the FCM procedure can be summarized as follows:

- Randomly initialize the degrees of membership, $F_{i,l}$, such that $\sum_{l}F_{i,l}=1,\;\forall i$.
- Determine the cluster centroids, $k_{l}\left[n\right]$, using the equation
  (2) $$k_{l}\left[n\right]=\frac{\sum_{i=1}^{I}\cdot F_{i,l}^{m}}{\sum_{i=1}^{l}\cdot F_{i,l}^{m}}.$$
- Calculate the Euclidean distance, $d_{i,l}^{2}$, of each data vector from each cluster centroid. Update the degree of membership of each data vector to each cluster, using the equation
  (3) $$F_{i,l}=\frac{d_{i,l}^{-2/(m-1)}}{\sum_{l=1}^{L}d_{i,l}^{-2/(m-1)}}.$$
- Go to the second step unless a predefined number of iterations have been reached or the change in the value of $F_{i,l}$ is negligible.

## 3. Application of the Combined Use of Acoustic Microscopy and Infrared Spectroscopic Mapping Imaging to the Investigation of Melanoma

### 3.1. Experimental Animal Model of Human Melanoma

**Figure 7:**
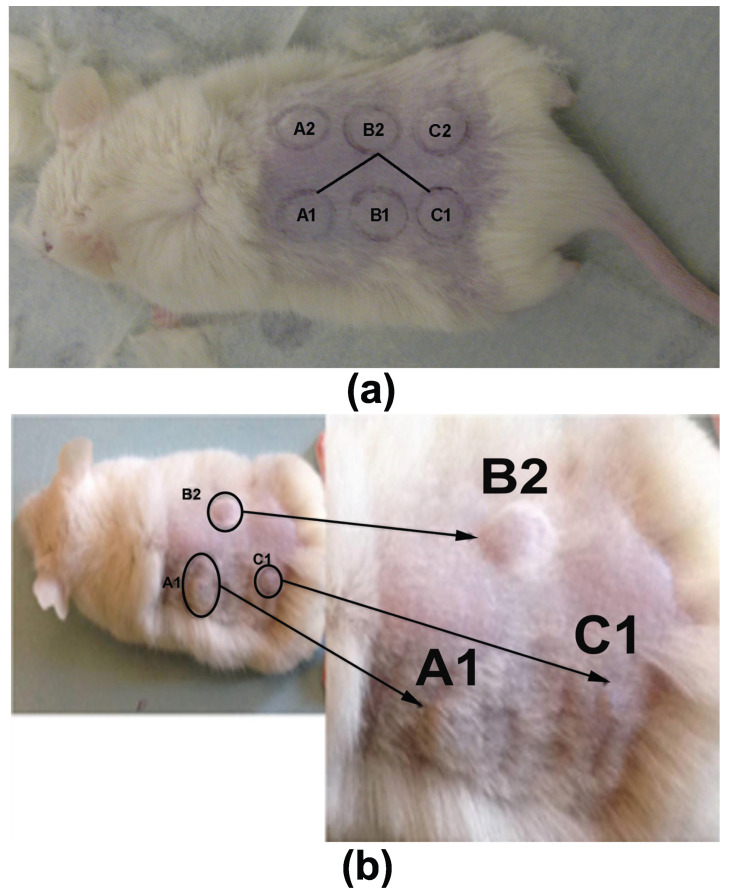
Mouse dorsal skin with (**a**) injection sites and (**b**) tumors at melanoma sites A1, B2 and C1 exhibiting gradual development that depended on the time post injection.

**Figure 8:**
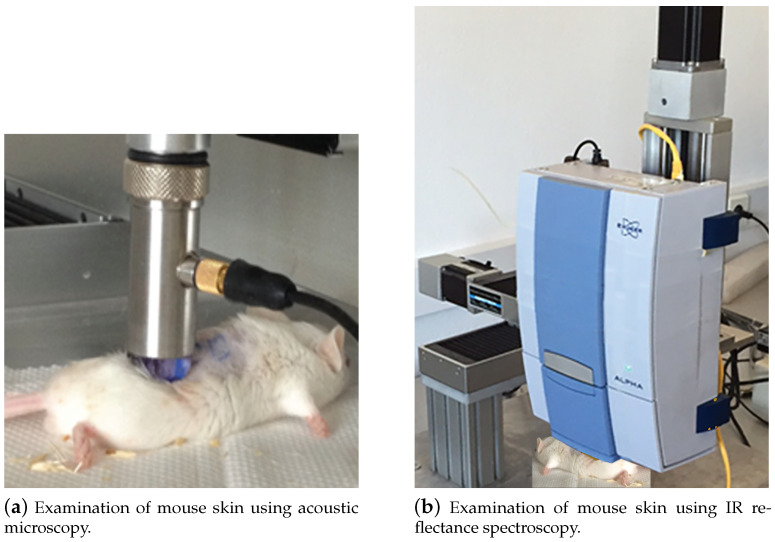
Examination of mouse skin using (**a**) acoustic microscopy and (**b**) IR reflectance spectroscopy.

Adult (90-day-old) NOD-SCID (NOD.CB17-${\mathrm{Prkdc}}^{\mathrm{scid}}$/NcrCrl; Harlan Laboratories, Italy) and SCID mice (n = 10) weighing 22–28 g were used. The mice were housed in the pathogen-free animal facility in the Anatomy, Histology and Embryology laboratory, Faculty of Veterinary Medicine, Aristotle University of Thessaloniki, which was maintained at 22 ± 1 °C and 60% humidity. They had a 12:12 h light/dark cycle and access to pellet food and water ad libitum, in accordance with the European Community Council directive 86/609/EEC. The experiment received approval from the Veterinary Directorate of Thessaloniki (approval number: 246867/2058-31.07.2014) and was in compliance with the guidelines of the Greek government and the Aristotle University Ethics Committee. All the procedures have been described previously [76]. Briefly, six injection sites were marked on the mouse’s back: A1, B1 and C1 on the left side and A2, B2 and C2 on the right. A total of 250 × $10^{3}$ SK-MEL-28 cells (Cell Lines Service, Eppelheim, Germany) in 20 DMEM (Dulbecco’s Modified Eagle’s Medium) per injection were endermally administered at three equally spaced dorsal skin sites, melanoma sites A1, B2 and C1, with a one week interval between the injections (Figure 7). Three contralateral sites with normal, non-injected tissue served as the controls (A2, B1, C2). The sites the most distant from each other were selected for injection, so that the developed tumors would not overlap. Administering three injections with one-week intervals between them provided tumors at different developmental stages in the same animal, thus minimizing the number of animals used. The fully developed tumors were evident based on direct observation and/or palpation, while early tumors like melanoma site B2 (M10B2) in mouse 10, for example, were not, and they were found using acoustic microscopy scans of the injection site. Five weeks after the first injection, the normal skin and tumor sites were visualized using acoustic microscopy (Figure 8a) and analyzed using IR spectroscopy (Figure 8b), in vivo, under deep anesthesia. Following the diagnostic investigation of the skin, the mice were sacrificed using a transcardial perfusion of 4% paraformaldehyde. The dorsal skin and organ targets for possible melanoma metastasis were removed.

The above-mentioned diagnostic techniques were also applied to the excised skin ex vivo. Six square skin pieces with a size of 5 × 5 mm or greater if necessary were cut from the excised dorsal skin from the three tumors and the contralateral control sites, dehydrated and embedded in paraffin. Sections were prepared for histological examination. Hematoxylin–Eosin (H&E) staining and immunohistochemistry for (a) DAPI ($4^{\prime }$,6-diamidino-2-phenylindole) and (b) monoclonal Mouse Anti-Human Melan-A (Dako, Glostrup, Denmark) revealed the structure and cytoarchitecture of the tumors and confirmed the development of skin melanoma cancer. Microphotographs of the histologically and immunohistochemically stained sections were acquired with a Nikon Eclipse C1 fluorescence microscope. The dimensions of the skin and tumors were calculated using Image Pro-Plus 6.3 software (media Cybernetics Inc., Rockville, MD, USA), supporting, besides other things, the decision regarding which frequencies would be used in the acoustic microscopy system.

### 3.2. Application of the Diagnostic Modalities

**Figure 9:**
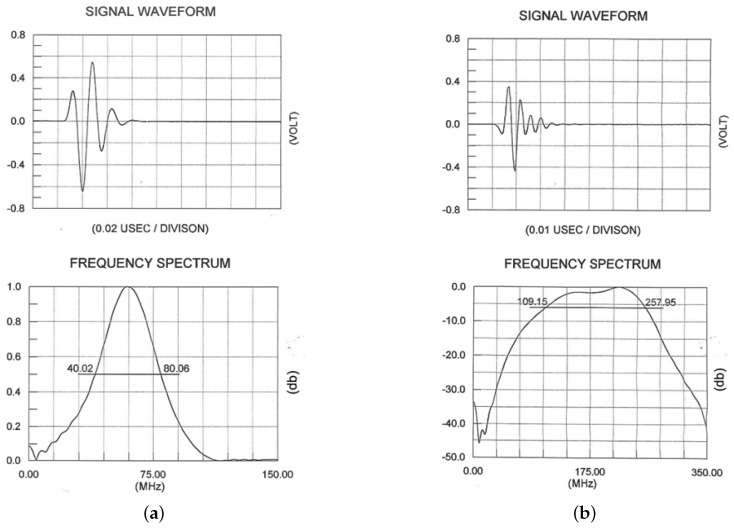
The signal waveforms and frequency spectra of the used transducers, (**a**) 60 MHz and (**b**) 175 MHz. The −6 db area is 40–80 MHz and 109–258 MHz spectral area for the 60 MHz and 175 MHz transducers respectively.

**Figure 10:**
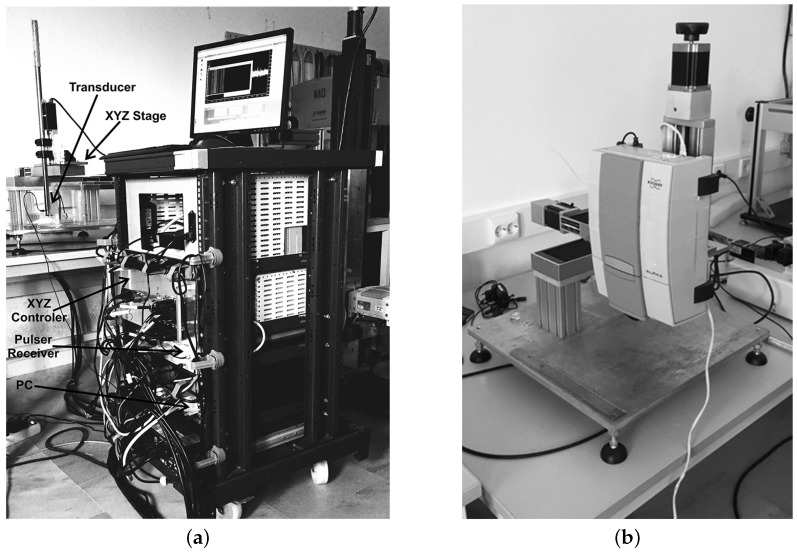
(**a**) Acoustic microscopy and (**b**) IR mapping setups.

The overall acoustic microscope system used in this work consisted of four basic components, i.e., custom-designed piezoelectric transducers (V3965 and PI75-1-R0.25, provided by Olympus), a pulser–receiver with a 400 MHz-bandwidth pre-amplifier (5910PR, from Panametrics NDT) and an analog-to-digital converter (ADC) (AL8xGTE-1.5, from Acquisition Logic). The transducers were broadband, with central operating frequencies of 60 MHz and 175 MHz and bandwidths of 40 MHz (40–80 MHz) and 140 MHz (111–243 MHz), respectively. The transducer operating at 60 MHz was the PI75-1-R0.25, which had a focal length of 5.7 mm and an element radius of 1.5 mm. The transducer operating at 175 MHz was the V3965, with a focal length of 5.9 mm and an element radius of 1.5 mm. The transducers’ impulse responses in the time and frequency domains are displayed in Figure 9a,b. The impulse response was provided by the transducer from Olympus, based on the ASTM E1065. Moreover, the pulser–receiver had a bandwidth of 400 MHz and was assisted by a pre-amplifier, which amplified the signals received from the transducer. The ADC sampled the analog input signal with an 8-bit resolution at a rate of up to 4.0 GSamples/s. The transducers were able to move to scan smaller or bigger areas of the melanoma using the previously described high-precision XYZ stages, with a smallest step of 0.1 μm. The probe of the acoustic microscope installed on the XYZ stage is displayed in Figure 10a,b.

The A-scan of the tumor’s internal structures is expected to be significantly weaker than the emitted pulse; hence, revealing small echoes in the A-scan signals is crucial for diagnosis. According to the simple model described above (Section 2.1), in each A-scan, we expected to detect signals that had characteristics well-localized in both frequency and time. Separating the different echoes and estimating the corresponding TOFs are challenging tasks, particularly when nearby echoes overlap.

**Figure 11:**
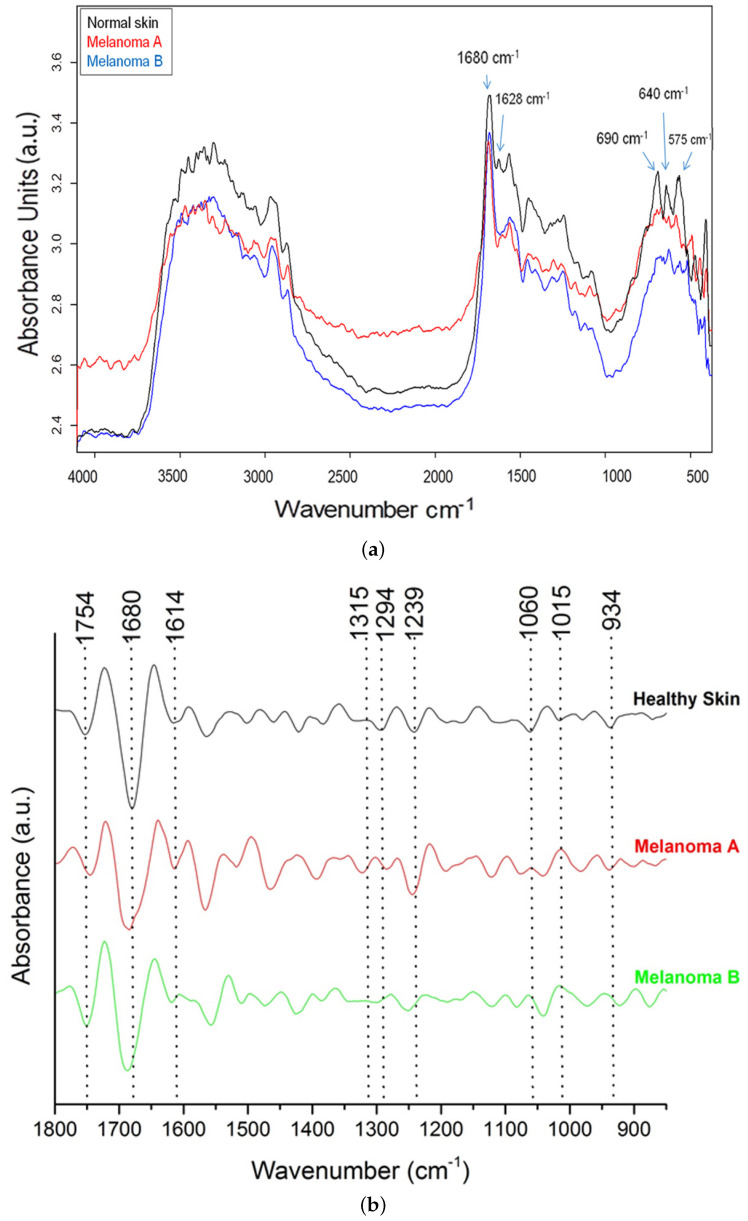
IR reflectance-mode spectra of healthy skin and two different melanoma sites, A1 and B2, of grafted mouse 2: (**a**) in the range of 4000–500 ${\mathrm{cm}}^{-1}$, (**b**) second-derivative spectra in the range of 1800–850 ${\mathrm{cm}}^{-1}$.

IR spectra of the tumor sites were acquired in vivo in the range of 7500–350 ${\mathrm{cm}}^{-1}$ and in reflectance mode. The obtained spectra and their second derivatives in the range of 4000–500${\mathrm{cm}}^{-1}$ are shown in Figure 11. Figure 11a displays the spectra of normal skin and melanoma A and B, and Figure 11b shows the derivative spectra. The most remarkable changes occurred in the range of 1800–850 ${\mathrm{cm}}^{-1}$ (Figure 11b). In the normal skin spectra, the maximum value for the asymmetric stretching band of $PO_{2}^{-}$ in the skin cells’ nucleic acids ($\nu_{asymPO_{2}}$) was found at 1239 ${\mathrm{cm}}^{-1}$ [77]. In the case of melanoma sites M2A1 and M2B2, this band shifted to 1246 ${\mathrm{cm}}^{-1}$ and 1250 ${\mathrm{cm}}^{-1}$. In addition, the symmetric stretching modes of $PO_{2}^{-}$ ($\nu_{symPO_{2}}$) were found at 1060 ${\mathrm{cm}}^{-1}$ (normal skin), 1076 ${\mathrm{cm}}^{-1}$ (M2A1) and 1079 ${\mathrm{cm}}^{-1}$ (M2B2). $PO_{2}^{-}$ is produced by the phosphodiesteric bonding that connects the nitrogenous bases and forms the primary structure of the DNA double helix. Additionally, the bands at 1015 ${\mathrm{cm}}^{-1}$ and 1060 ${\mathrm{cm}}^{-1}$ in the normal skin spectra, which were attributed to the stretching vibration of the C-O bond in deoxyribose in the nucleic acids, were reduced in both the melanoma spectra (Figure 11b). These results are in accordance with those in the literature [50]. Hydrogen bonds (H-bonding) between the water and DNA molecules could be detected based on a wide band at 3400 ${\mathrm{cm}}^{-1}$. Also, the bands at 1680 ${\mathrm{cm}}^{-1}$ and 2600 ${\mathrm{cm}}^{-1}$ were attributed to the H-bonding absorbance [77]. In particular, the bands in the melanoma spectra (M2A1 and M2B2) at 1680 ${\mathrm{cm}}^{-1}$ were shifted.

As shown in Figure 11b, the intensity of the bands at 1648 ${\mathrm{cm}}^{-1}$ and 934 ${\mathrm{cm}}^{-1}$ (corresponding to the a-helix of DNA) was lower in the melanoma A and B spectra than the normal skin spectra [78]. Assuming that the H-bonding absorbance changes in the case of cancer, differences in the absorbance intensity or shifts in the bands of the H-bonds in the IR spectra, as shown in Figure 11, could constitute markers. So, our finding of differentiation in specific bands between the spectra of normal tissue and melanoma M2A1 or M2B2 demonstrates that IR can offer valuable information about normal skin and melanoma. Which bands are differentiated from those in the normal skin spectra and which ones are reduced depends on additional parameters, such as the progress or type of the tumor. All the aboved described in the previous two paragraphs are summarized in Table 1.

**Figure 12:**
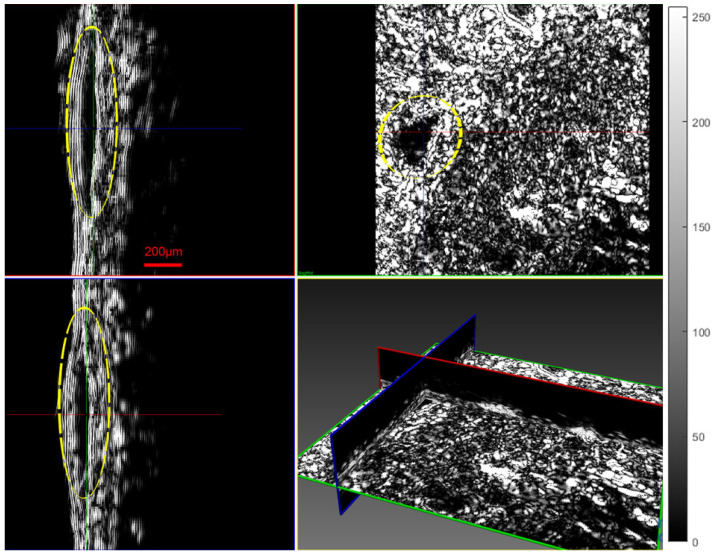
Three-dimensional visualization of tumor M10B2 using acoustic microscopy (tumor thickness of 66.6 µm). The scale line, in red, represents 200 µm. The dashed yellow line indicates the area of the tumor.

The probes in both systems used the same XYZ moving stages, provided by Aerotech. The step resolution of the X-Y stages was 100 nm. The acoustic microscopy and infrared spectroscopic probes were mounted on the same Z stage. The focal length of the ultrasonic probe transducer for insonification was 20 µm. The light spot area of the infrared probe was 0.8 mm. The infrared spectrometer was at a fixed offset distance from the center of the measurement spot area. During the procedure, the initial measurement point was determined accurately for both systems based on its distance from the home position of the XY stage. At the beginning of the measurement using the infrared device, the XY stage was moved the offset distance from its home position, and then the measurement started from the same point as the acoustic microscopy. The fact that the step resolution was 0.1 µm, which was 10 times less than the resolution of the provided images, meant that a possible distance deviation error did not affect the efficiency of the method. The final result of the scanning procedure, using the XYZ moving stages, is the B-scan and C-scan tomographic images, displayed in Figure 12 in which a starting developing tumor with a cross section thickness of 66.6 µm was revealed.

### 3.3. System Evaluation and Results

The animal model developed in this study was the first in which human malignant melanoma cells were endermally grafted, aiming to mimic the malignant melanomas that develop in humans. Nine out of the ten grafted mice developed tumors at the injection sites, appearing 2–3 weeks post injection. Both modalities were applied to scan the tumor sites in vivo, demonstrating their potential to identify melanoma tumors at different developmental stages.

#### 3.3.1. Histological Results

**Figure 13:**
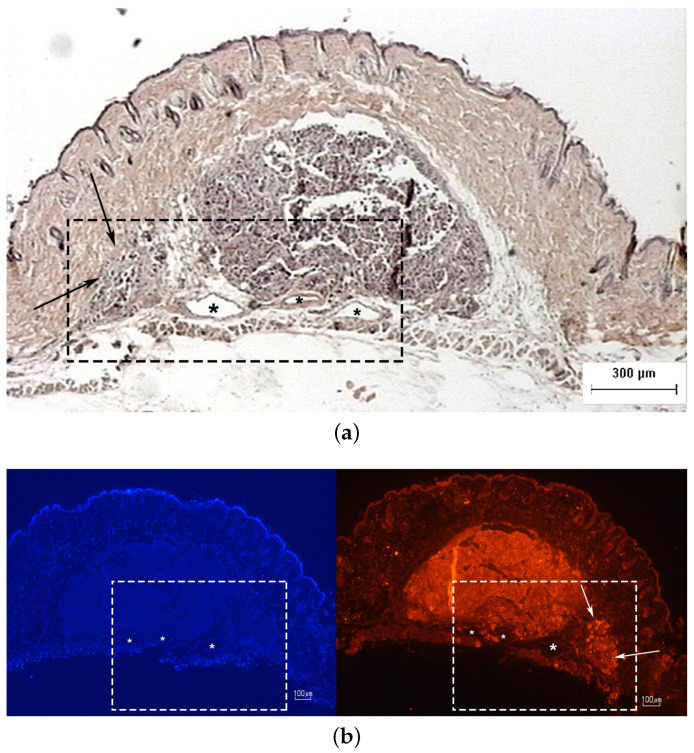
Photomicrographs of melanoma site M5B2 confirming the presence of malignant melanocytes: (**a**) an H&E-stained section and (**b**) an adjacent section immunostained with DAPI (blue) and Melan A (red). Arrows indicate tumor protrusions and asterisks indicate the presence of the characteristic triad of vessels.

Histological examination of Hematoxylin–Eosin (H&E)-stained sections from the scanned tumors showed that all the tumors represented early lesions, with neoplastic melanocytes confined within the skin. The tumors examined displayed nested, lentil-shaped accumulations of atypical melanocytes, which extended from the epidermis to the dermis without crossing the histological borders of the dermis (Figure 13a).

Immunohistochemistry is a reliable tool to distinguish melanomas from other tumors. Melan A (Protein Melan-A or Melanoma Antigen Recognized by T Cells 1, MART-1) is a melanocytic lineage antigen that is present in melanin-producing skin cells. It is selectively expressed in most primary and metastatic melanomas and is therefore considered a reliable melanocytic marker. Immunohistochemical analysis of sections from the scanned tumors revealed that they consisted of nucleated cells (marked with 4,6-diamidino-2-phenylindole, DAPI) that were all positive for Melan A, confirming the presence of malignant melanoma (Figure 13b).

#### 3.3.2. Acoustic Microscopy Results

**Figure 14:**
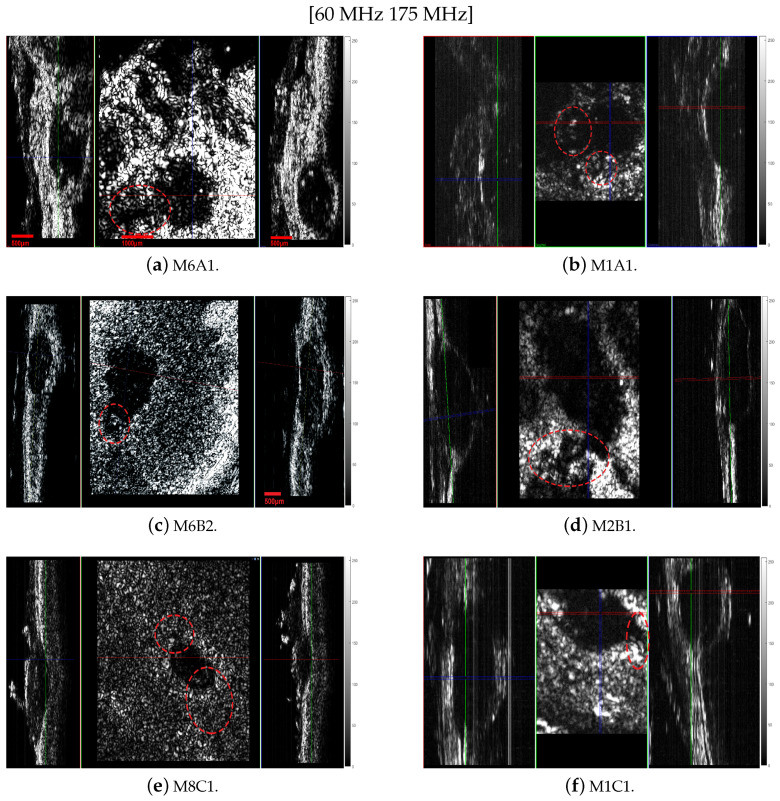
Tomographic images obtained using acoustic microscopy. The left column consists of images acquired using the 60 MHz transducer, whereas the images on the right were obtained with the 175 MHz transducer. The first row consists of images of melanoma site A, the second of melanoma site B and the third of melanoma site C. Every image is separated into three parts representing, from left to right, the sagittal, coronal and axial sections. The resolution when using the 175 MHz transducer was 5 µm in the coronal and axial images and 20 µm in the sagittal images, and when using the 60 MHz transducer, it was 12.5 µm in the coronal and axial images and 20 µm in the sagittal images.

The selection of an operating frequency is based on a trade-off between the attenuation of a reflected wave and the resolution of the developing tumor’s cytoarchitecture. Specifically, higher-frequency transducers provide a higher resolution but more attenuation, i.e., a lower penetration depth. Hence, the selected transducers supplied complementary information, meaning the 60 MHz transducer had a greater penetration depth, whereas the 175 MHz one had a higher resolution. In the following subsection, we describe the results obtained using the above-mentioned transducers: The acoustic microscopy images displayed in Figure 14 show the system’s capability to detect small, early-stage melanoma tumors at the scale of hundreds of µm. Specifically, the resolution obtained using the 175 MHz transducer was 5 µm in the coronal and axial images and 20 µm in the sagittal ones, and that obtained using the 60 MHz transducer was 12.5 µm in the coronal and axial images and 20 µm in the sagittal ones. The depth of penetration of the skin and tumors when using the 60 MHz transducer was 2.5 mm, and it was 1.2 mm when using the 175 MHz one. This was a result of the high attenuation of the ultrasound waves in the skin structure at the used frequencies. Consequently, the images in these ultrasound frequency ranges had a low signal-to-noise ratio, especially those obtained with the 175 MHz transducer. The images obtained using the 60 MHz transducer were significantly improved even though the resolution was lower. Yet the hypoechoic response of the tumor, compared to that of the normal skin, was evident in all of the images regardless of how they were obtained, permitting the detection of the melanoma. This is a significant advantage since the normal lesions and the skin were not totally hypoechoic, as in the case of all the melanoma images. Moreover, the borders of the cancerous area were distinct, and in many cases the displacement of the nearby tissue was evident (Figure 14e, M8C1).

**Figure 15:**
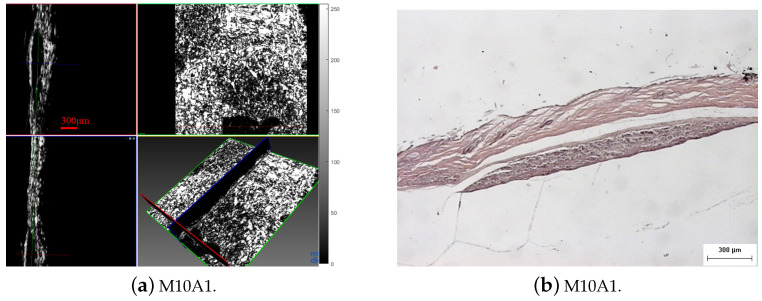
Tumor M10A based on (**a**) Acoustic microscopy 3D visualization (tumor thickness 296 µm (measurement line) and (**b**) H-E stained histological section.

**Figure 16:**
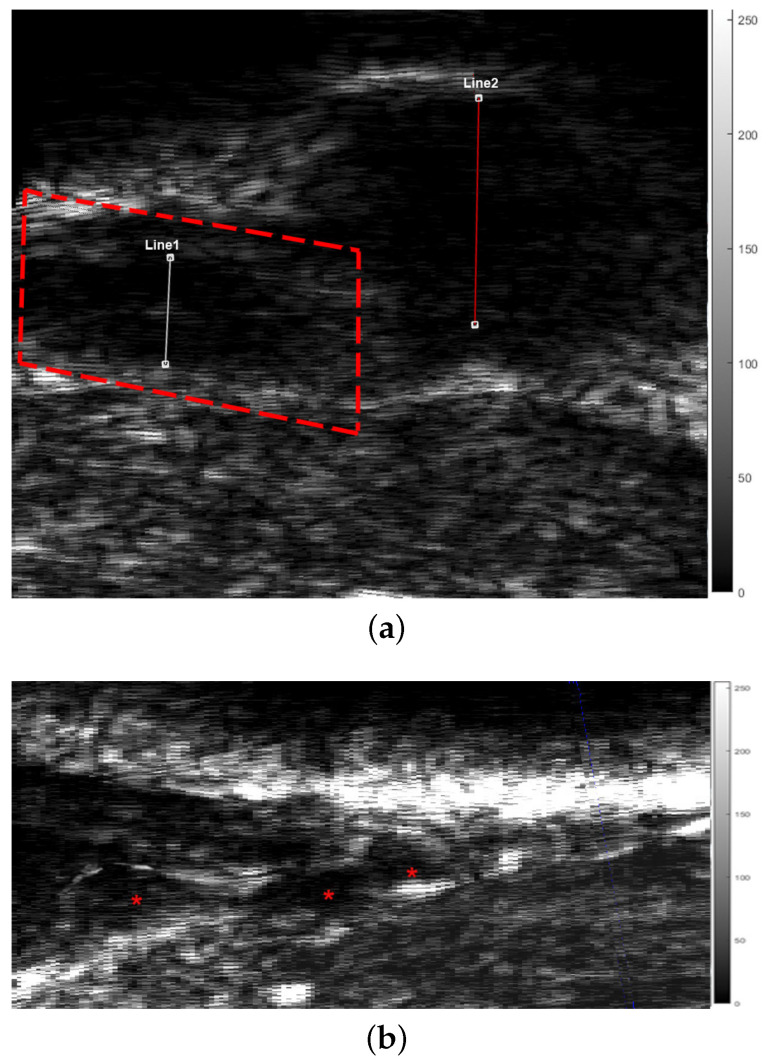
Acoustic microscopy B-scan (coronal section) of tumor M5B2 revealing (**a**) finger like protrusions (arrows) and (**b**) triad of vessels (angiogenesis) (asterisks). Image (**b**) magnification of the dotted frame in image (**a**). Line 1 = 629 µm and Line 2 = 296 µm. The corresponding histological and immunohistochemical images are presented in Figure 13.

The characteristics of the proposed acoustic microscopy system allowed us to observe early tumors, infiltration of the surrounding tissue and signs of angiogenesis (Figure 14, especially in Figure 14c–e (marked with red dotted circles). Early detection of small tumors was possible in the cases of tumor M8C1 (Figure 14e) in mouse 8 and tumors M10A1 (Figure 15) and M10B2 (Figure 12)) in mouse 10. The 296 µm tumor was detected using both the modalities. The dimensions of the tumor were correlated with the dimensions revealed by the histological examination (Figure 15). An even earlier tumor, nondetectable with in vivo palpation and with a height of only 66.6 µm, was detected and visualized in M10B2 (Figure 12). Infiltration of the surrounding tissue (Figure 16a) was visualized and observed in many cases. Another significant finding was the presence of a triad of parallel vessels in the deeper layers of the tumor, representing the inauguration of angiogenesis (Figure 16b). Angiogenesis vessels with a diameter of 30–60 µm were revealed. The accuracy of visualization of the infiltration and vessels and their topography using the proposed acoustic microscopy approach was cross-checked using the corresponding histological and immunohistochemical images of the same tumors (Figure 13a,b). These findings from both the acoustic microscopy and histological images were also in very good agreement with the related research. Table 2 shows the days post SKMEL28 cell injection when scanning and excision took place, as well as the dimensions of the tumors that developed (max width, min width and height), measured using the acoustic microscopy and the spectroscopic mapping images.

#### 3.3.3. IR Spectroscopic Mapping Results

**Figure 17:**
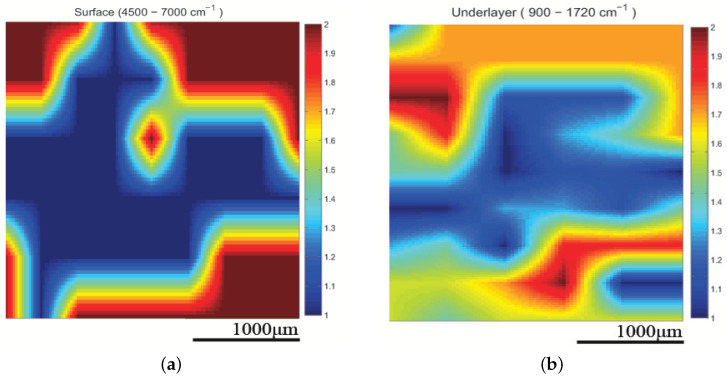
Spectroscopic mapping images of tumor M10A1 (**a**) at surface and (**b**) in deeper layers. Blue corresponds to malignant melanoma and red corresponds to healthy tissue. Corresponding images of tumor obtained using acoustic microscopy shown in Figure 15a and Figure 22.

**Figure 18:**
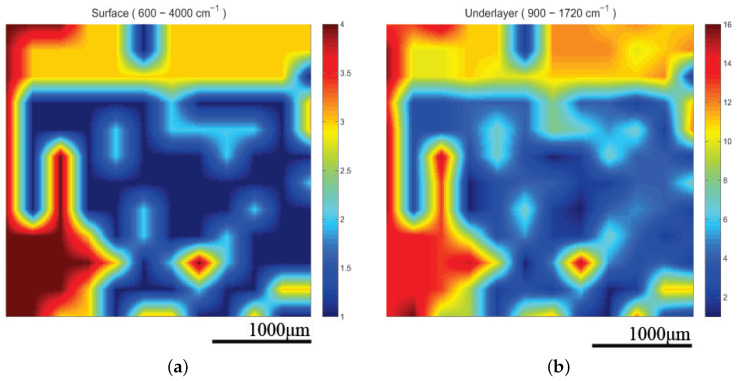
Spectroscopic mapping images of tumor M2A1 (**a**) at surface and (**b**) in deeper layers. Blue corresponds to malignant melanoma and red corresponds to healthy tissue. Corresponding acoustic microscopy images obtained using 175 MHz transducer appear in Figure 14 (M2A1).

**Figure 19:**
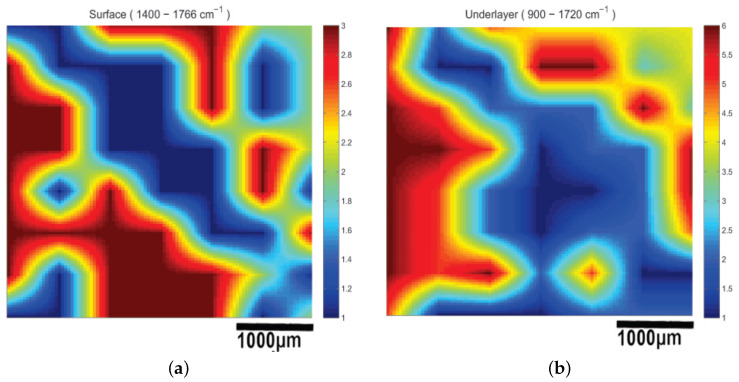
Spectroscopic mapping images of tumor M2B1 (**a**) at surface and (**b**) in deeper layers. Blue corresponds to malignant melanoma and red corresponds to healthy tissue. Corresponding acoustic microscopy image obtained using 175 MHz transducer appears in Figure 14 (M2B1).

**Figure 20:**
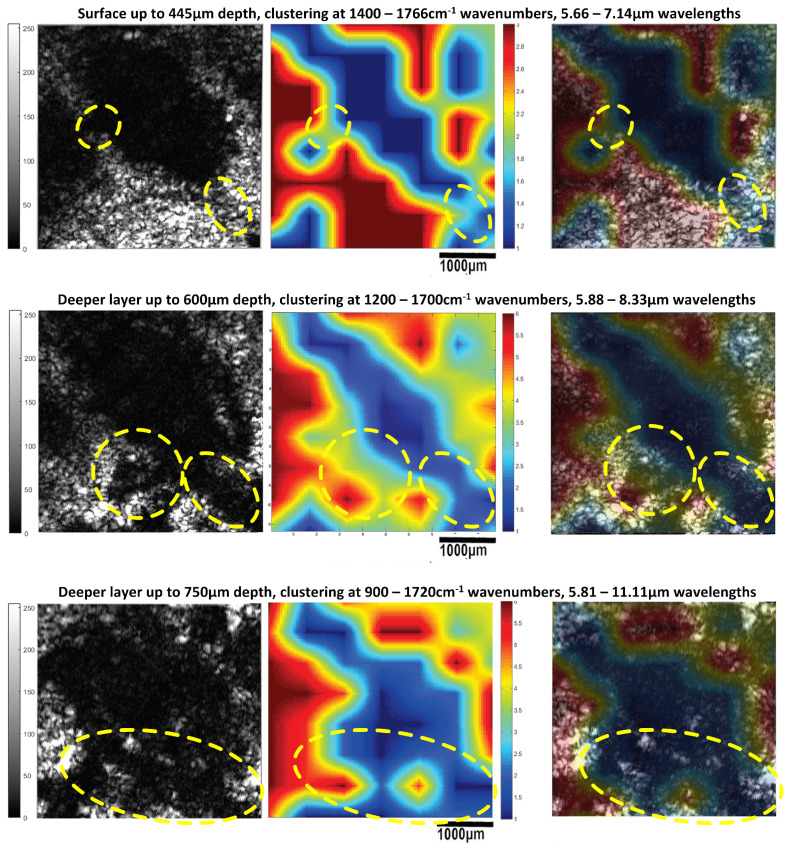
Sagittal sections of ultrasonic 3D images presenting the development of a melanoma tumor in mouse 3 at varying depths, obtained using both modalities (acoustic microscope and infrared spectroscope). The blue areas in the mapping images and black areas in the ultrasonic images correspond to malignant melanoma, and the red areas in the mapping images and white areas in the ultrasonic images correspond to healthy tissue.

**Figure 21:**
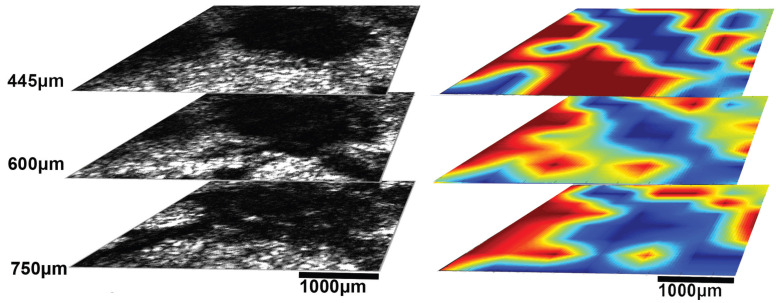
Sagittal sections of ultrasonic 3D images presenting the development of a melanoma tumor in mouse 3 at varying depths, obtained using both modalities (acoustic microscope and infrared spectroscope) (see also Figure 20). The blue areas in the mapping images and black areas in the ultrasonic images correspond to malignant melanoma, and the red areas in the mapping images and white areas in the ultrasonic images correspond to healthy tissue.

The infrared spectrometer used was the Bruker model Alpha with an S/N:101119, which was mounted to the XYZ stage in order to acquire an array of reflectance spectra corresponding to the Region Of Interest (ROI). The setup of the IR mapping device is illustrated in Figure 10b. During the application of the two modalities, multiple IR spectra of the same samples were obtained to assess the sensitivity of the IR spectroscopy and evaluate which would be the most useful setup for future diagnostic applications in the crucial spectral range described in Section 3.2. The IR spectroscopic mapping images of two tumor sites are shown in Figure 17, Figure 18 and Figure 19. The dimensions of the array of the acquired reflectance spectra depended on the dimensions of the lesion and very small overlaps of the light spot areas in the spectra between the measurements of different regions. Pseudocolors were used in the images to classify the spectra that belonged to each cluster. The number of clusters is indicated on the colorbars on the right of every image. The infrared mapping and ultrasonic/acoustic microscopy images were in very good agreement (Figure 20 and Figure 21) at the surface and across the whole depth of the tumors. It is evident from these results that the infrared radiation penetrated the whole tumor and skin structure, obtaining biochemical information from all the superimposed layers and allowing us to draw conclusions from this. In conclusion, distinct differentiation between the tumor and healthy skin areas was achieved due to the differentiation between the spectra (Section 3.2) in the reflection mode and the signal processing procedure (Section 2.3). The high degree of agreement between the spectroscopic mapping and acoustic microscopy images validate the spectroscopic information, and these images’ repeated agreement with the corresponding histological results also demonstrates the success of this method. Moreover, the figures provided in Table 3 are also applicable to the IR spectroscopic mapping images.

**Figure 22:**
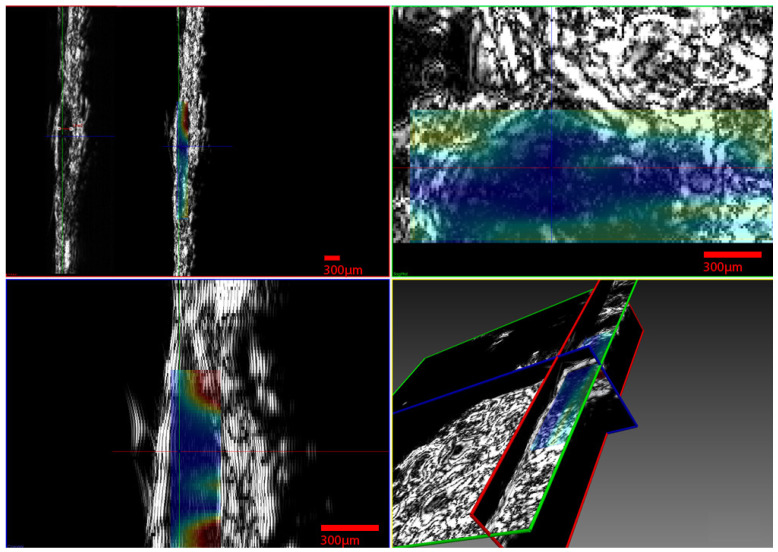
Fusion of the results for tumor M10A1 acquired using acoustic microscopy and spectroscopic mapping imaging from 900 ${\mathrm{cm}}^{-1}$ to 1766 ${\mathrm{cm}}^{-1}$ (Figure 17). The thickness of the tumor is 296 µm (at the measurement line). A corresponding image of the histological examination results is shown in Figure 15.

## 4. Discussion

**Figure 23:**
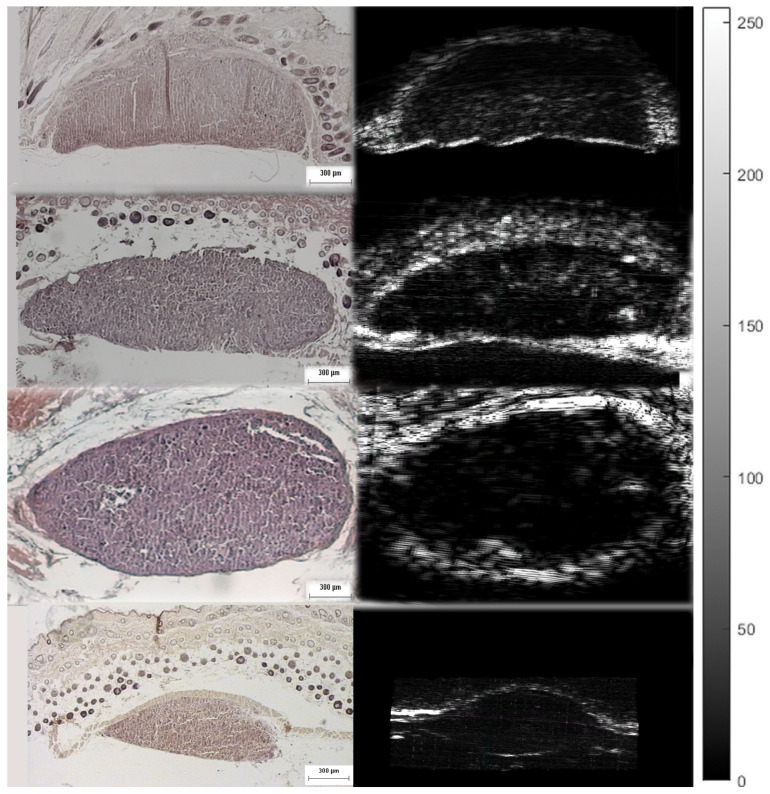
Ultrasonic/acoustic microscopy slices and the corresponding histological analysis images, showing the accordance between them.

The tumors were hypoechoic compared to the normal skin. This was observed in every tumor examined, regardless of the tumor’s development. The hypoechoic tumors exhibited a distinct IR spectrum which was clearly differentiated in certain aspects from that of the normal skin. The proposed combination of the two modalities provided a high-resolution reproduction of the tumor morphology, found to be in absolute accordance with the results of the histological analysis (Figure 15, Figure 17, Figure 18 and Figure 19, Figure 21 and Figure 23). The complete representation of the tumor morphology permits the observation of certain pathognomonic characteristics, which may lead to the early and accurate non-invasive diagnosis of melanoma. More specifically, acoustic microscopy applied to mouse skin permitted the acquisition of high-resolution images extending up to hundreds of µm in depth. Hence, the features of early melanoma tumor development were visualized. The tumors’ shapes and dimensions correlated completely with the measurements acquired from the histological assessment (Figure 13, Figure 15 and Figure 23).

Furthermore, finger-like protrusions from the tumors and triads of new vessels (possibly an artery, vein and lymphatic vessel, characteristic of tumor angiogenesis) penetrating them, features indicating malignancy and metastatic tendencies, were also revealed (Figure 13, Figure 14, Figure 15 and Figure 16). Angiogenesis in particular was revealed using both the devices and methods (Figure 20 and Figure 21). The IR spectroscopic mapping results may be explained by variation in the H-bonding in melanocytic cells. This finding is in accordance with others for breast and oral cavity cancers, where variation in the H-bonding and hence the blue shift in the C-OH band have been attributed to an increase in protein phosphorylation during cancer cell proliferation [76,79,80]. In other literature reports [80,81], significant cellular activity in malignant tumors may be demonstrated by the absence of an evident DNA band. In the present work, only the H-bonding peak shift and deoxyribose peak intensity reduction were recorded. Consequently, the evolution of the tumor or melanoma and the stage of the cancer might play a crucial role in IR classification.

Besides the biochemical findings described above, the advantages of combining the two modalities and their complementarity are evident: the cluster images were taken at different wavelengths, taking into consideration not only the biochemical analysis method and findings in Table 1 but also the electromagnetic and photonics theory regarding the increase in the penetration depth with an increasing wavelength (see the related description in the fourth paragraph of the Introduction) or a decreasing absorption coefficient [82]. Based on this extensive research, the wavelengths used to cluster the mapping slices at various depths were chosen, and when the wavelengths increased, the penetration depth increased, and the distribution of the blue areas (malignacy) in the mapping clustering slices matched that of the hypoechoic areas in the ultrasonic slices, as shown by the maximal agreement between them. This is very well displayed in Figure 20 and Figure 21, where when the wavelengths chosen increased, the clustering mappings were in accordance with the deeper slices of the ultrasound, validating the high fidelity of the information provided by both the modalities. It is also very interesting that even in the areas where the malignancy was not so hypoechoic in the ultrasonic slices, the color in the heatmap slices obtained using the infrared modality was not as strong a blue as in the totally hypoechoic malignancy areas of the ultrasonic ones. While the depth increased and the angiogenesis described before was revealed in the ultrasonic slices, clusters were also revealed with high accuracy in the infrared slices when higher wavelengths were used. All of this is depicted in Figure 20 and Figure 21 with distinct yellow lines.

In conclusion, the images acquired using both the proposed modalities correlated completely not only with each other (Figure 20, Figure 21 and Figure 22) but also with the acquired histological and immunohistochemical data (Figure 13, Figure 14, Figure 15, Figure 16 and Figure 17).

## 5. Conclusions

In this work, acoustic microscopy and IR spectroscopy were combined into an in situ diagnostic system for common melanoma. On one hand, acoustic microscopy using high-frequency transducers (>40 MHz) provides high-resolution images of skin regions, so morphological information regarding small-sized melanomas (tens of micrometers) can be derived with a resolution of 5 µm. On the other hand, matrices of IR spectra from an ROI can be used for IR mapping imaging using a proper clustering algorithm. These IR images map the differential distribution of biochemical changes in healthy skin and skin with a developing melanoma. The proposed system was applied to melanoma tumors that developed in immunodeficient mice injected endermally with human malignant melanoma cells. The present results demonstrate the ability of the implemented methods to detect melanoma tumors at the early stages of their development (less than three weeks post injection). Moreover, the identification of vessel triads (angiogenesis) may prove to be a pathognomonic indicator for the diagnosis of malignant tumors [83].

Further research is required to assess the biochemical changes and corresponding chemical bands and their components during tumor growth, optimizing the FTIR mapping images while not affecting the complexity of the method/system. Considering that both the modalities used in the present study produced consistent and promising results, our aim for the future is the development of a probe combining acoustic microscopy and IR spectroscopy capabilities, which could be easily applied as a diagnostic tool for skin lesions with suspected malignancy. Moreover, even though the infrared mapping images provided very good results, the pure spectra acquired from the melanoma will be repeatedly and extensively studied, targeting the extraction of more detailed information on the biomolecules existing in its structure. Lastly, the procedure for fusing the malignacy areas identified using both the modalities with an increasing wavelength will be automated using pattern recognition techniques.

## Figures and Tables

**Table 1:** Differences in absorbance intensity and wavenumber shifts in the IR spectra in Figure 6.

	Normal Skin	Melanoma A	Melanoma B	Literature
$\nu_{asymPO_{2}}$	1239 ${\mathrm{cm}}^{-1}$	1246 ${\mathrm{cm}}^{-1}$	1250 ${\mathrm{cm}}^{-1}$	[77]
$\nu_{symPO_{2}}$	1060 ${\mathrm{cm}}^{-1}$	1076 ${\mathrm{cm}}^{-1}$	1079 ${\mathrm{cm}}^{-1}$	
a-helix DNA	1648 ${\mathrm{cm}}^{-1}$ and 934 ${\mathrm{cm}}^{-1}$	Decreased at 1648 ${\mathrm{cm}}^{-1}$ and 934 ${\mathrm{cm}}^{-1}$	Decreased at 1648 ${\mathrm{cm}}^{-1}$ and 934 ${\mathrm{cm}}^{-1}$	[78]
Stretching vibration of C-O bond in deoxyribose in nucleic acids	1015 ${\mathrm{cm}}^{-1}$ and 1060 ${\mathrm{cm}}^{-1}$	Reduced at 1015 ${\mathrm{cm}}^{-1}$	Reduced at 1015 ${\mathrm{cm}}^{-1}$	[50]
H-bonding absorbance	1680 ${\mathrm{cm}}^{-1}$ and 2600 ${\mathrm{cm}}^{-1}$	Band at 1680 ${\mathrm{cm}}^{-1}$ is shifted	Band at 1680 ${\mathrm{cm}}^{-1}$ is shifted	H-bonding changes in case of cancer

**Table 2:** Tumors’ dimensions acquired from the tomographic acoustic microscopy and infrared spectroscopic mapping images.

Tumors (A1, B2, C1)												
**Days Taken Post Engraftment for the Development of the Tumor**						**Dimensions in mm: D1 (Max Width), D2** **(Min Width) and H (Height)**						
	A1	B2	C1		A1			B2			C1	
				D1 (mm)	D2 (mm)	Height	D1 (mm)	D2 (mm)	Height	D1 (mm)	D2 (mm)	Height
Mouse 2	49	41	34	3.4	3.3	0.7777				2	1.18	0.6314
Mouse 3	35	15		3.55	1.89	0.7623	3.73	1.89	0.7931			
Mouse 4	40	34		5.28	4.1	1.4861	4.18	3.53	1.4168			
Mouse 5	34	23	18	4.1	2.29	1.078	2.6	1.26	0.7623			
Mouse 6		29	24				2.47	1.21	0.7546	2.12	1.26	0.4543
Mouse 7	40	35	30	1.8	1.55	0.8547	2.7	1.76	0.6853	2.55	1.53	0.3696
Mouse 8	34		19	3.43	2.62	1.2936				2.83	2.75	1.078
Mouse 9			17							3.1	1.4	0.6622
Mouse 10	25	17	10	1.4	2.87	0.2695	1.32	1.29	0.0693	1	0.39	0.0666

**Table 3:** Wavelength–wavenumber clustering band numbers (Figure 20 and Figure 21).

Wavelength–Wavenumber Clustering Band Numbers (Figure 20 and Figure 21)				
**Area**	**Wavenumbers**		**Wavelengths**	
	${\mathrm{cm}}^{-1}$	${\mathrm{cm}}^{-1}$	µm	µm
Surface to 445 µm	1400	1766	5.66	7.14
Deeper layer at 600 µm	1200	1700	5.88	8.33
Deeper layer at 750 µm	900	1720	5.81	11.11

## Data Availability

The data presented in this study are available in this article.

## References

[1] Siegel R.L., Miller K.D., Wagle N.S., Jemal A. (2023). Cancer statistics, 2023. CA Cancer J. Clin..

[2] Siegel R.L., Giaquinto A.N., Jemal A. (2024). Cancer statistics, 2024. CA Cancer J. Clin..

[3] Bolick N.L., Geller A.C. (2021). Epidemiology of Melanoma. Hematol. Clin. N. Am..

[4] Ahmed B., Qadir M.I., Ghafoor S. (2020). Malignant Melanoma: Skin Cancer—Diagnosis, Prevention, and Treatment. Crit. Rev. Eukaryot. Gene Expr..

[5] Tímár J., Ladányi A. (2022). Molecular Pathology of Skin Melanoma: Epidemiology, Differential Diagnostics, Prognosis and Therapy Prediction. Int. J. Mol. Sci..

[6] Catalano O., Corvino A. (2023). Ultrasound of skin cancer: What we need to know. Seminars in Ultrasound, CT and MRI.

[7] Siegel R., Naishadham D., Jemal A. (2012). Cancer statistics, 2012. CA Cancer J. Clin..

[8] Linos E., Swetter S.M., Cockburn M.G., Colditz G.A., Clarke C.A. (2009). Increasing burden of melanoma in the United States. J. Investig. Dermatol..

[9] Rigel D.S. (2010). Epidemiology of melanoma. Seminars in Cutaneous Medicine and Surgery.

[10] Zaharna M., Brodell R.T. (2003). It’s time for a “change” in our approach to early detection of malignant melanoma. Clin. Dermatol..

[11] Ruocco E., Argenziano G., Pellacani G., Seidenari S. (2004). Noninvasive imaging of skin tumors. Dermatol. Surg..

[12] Foster F.S., Lockwood G., Ryan L., Harasiewicz K., Berube L., Rauth A. (1993). Principles and applications of ultrasound backscatter microscopy. IEEE Trans. Ultrason. Ferroelectr. Freq. Control.

[13] Semple J.L., Gupta A.K., From L., Harasiewicz K.A., Sauder D.N., Foster F.S., Turnbull D.H. (1995). Does High-Frequency (40–60 MHz) Ultrasound Imaging Play a Role in the Clinical Management of Cutaneous Melanoma?. Ann. Plast. Surg..

[14] Passmann C., Ermert H. (1996). A 100-MHz ultrasound imaging system for dermatologic and ophthalmologic diagnostics. IEEE Trans. Ultrason. Ferroelectr. Freq. Control.

[15] El Gammal S., El Gammal C., Kaspar K., Pieck C., Altmeyer P., Vogt M., Ermert H. (1999). Sonography of the Skin at 100 MHz Enables In Vivo Visualization of Stratum Corneum and Viable Epidermis in Palmar Skin and Psoriatic Plaquesy1. J. Investig. Dermatol..

[16] Knspik D., Starkoski B., Pavlin C.J., Foster F.S. (2000). A 100–200 MHz ultrasound biomicroscope. IEEE Trans. Ultrason. Ferroelectr. Freq. Control.

[17] Strohm E.M., Kolios M.C. (2009). Measuring the mechanical properties of cells using acoustic microscopy. Proceedings of the Annual International Conference of the IEEE Engineering in Medicine and Biology Society, EMBC 2009.

[18] Oh J.T., Li M.L., Zhang H.F., Maslov K., Stoica G., Wang L.V. (2006). Three-dimensional imaging of skin melanoma in vivo by dual-wavelength photoacoustic microscopy. J. Biomed. Opt..

[19] Zhang H.F., Maslov K., Stoica G., Wang L.V. (2006). Functional photoacoustic microscopy for high-resolution and noninvasive in vivo imaging. Nat. Biotechnol..

[20] Ntziachristos V., Razansky D. (2010). Molecular imaging by means of multispectral optoacoustic tomography (MSOT). Chem. Rev..

[21] McCormack D.R., Bhattacharyya K., Kannan R., Katti K., Viator J.A. (2011). Enhanced photoacoustic detection of melanoma cells using gold nanoparticles. Lasers Surg. Med..

[22] Youssef S., Seviaryna I., Shum D., Maeva E., Malyarenko E., Rahman N., Maev R.G. (2019). High-resolution quantitative acoustic microscopy of cutaneous carcinoma and melanoma: Comparison with histology. Ski. Res. Technol..

[23] Fedorov Kukk A., Wu D., Gaffal E., Panzer R., Emmert S., Roth B. (2022). Multimodal system for optical biopsy of melanoma with integrated ultrasound, optical coherence tomography and Raman spectroscopy. J. Biophotonics.

[24] Kratkiewicz K., Manwar R., Rajabi-Estarabadi A., Fakhoury J., Meiliute J., Daveluy S., Mehregan D., Avanaki K.M. (2019). Photoacoustic/Ultrasound/Optical Coherence Tomography Evaluation of Melanoma Lesion and Healthy Skin in a Swine Model. Sensors.

[25] Kukk A.F., Wu D., Gaffal E., Panzer R., Emmert S., Roth B., Azar F.S., Intes X., Fang Q. (2023). Multimodal imaging system with ultrasound, photoacoustics, and optical coherence tomography for optical biopsy of melanoma. Multimodal Biomedical Imaging XVIII.

[26] Karagiannis T., Athanassopoulos E., Amanatiadis S., Karagiannis G.T., Campagnola P.J., Maitland K.C., Roblyer D.M. (2021). Design and operation analysis of a fabricated spectracoustic probe for tissue classification in microscopic level. Multiscale Imaging and Spectroscopy II.

[27] Bezugly A., Rembielak A. (2021). The use of high frequency skin ultrasound in non-melanoma skin cancer. J. Contemp. Brachytherapy.

[28] Belfiore M.P., Reginelli A., Russo A., Russo G.M., Rocco M.P., Moscarella E., Ferrante M., Sica A., Grassi R., Cappabianca S. (2021). Usefulness of High-Frequency Ultrasonography in the Diagnosis of Melanoma: Mini Review. Front. Oncol..

[29] Wang C., Guo L., Wang G., Ye T., Wang B., Xiao J., Liu X. (2021). In-vivo imaging of melanoma with simultaneous dual-wavelength acoustic-resolution-based photoacoustic/ultrasound microscopy. Appl. Opt..

[30] Kukk A.F., Scheling F., Panzer R., Emmert S., Roth B. (2024). Non-invasive 3D imaging of human melanocytic lesions by combined ultrasound and photoacoustic tomography: A pilot study. Sci. Rep..

[31] Kukk A.F., Scheling F., Panzer R., Emmert S., Roth B. (2023). Combined ultrasound and photoacoustic C-mode imaging system for skin lesion assessment. Sci. Rep..

[32] Park J.H., Lee B.C., Lee B.C., Seo Y.C., Kim J.H., Kim D.J., Lee H.J., Moon H., Lee S. (2023). Drug delivery by sonosensitive liposome and microbubble with acoustic-lens attached ultrasound: An in vivo feasibility study in a murine melanoma model. Sci. Rep..

[33] Zhang F., Niu G., Lu G., Chen X. (2011). Preclinical lymphatic imaging. Mol. Imaging Biol..

[34] Lavarello R.J., Ridgway W.R., Sarwate S.S., Oelzeb M.L. (2013). Characterization of thyroid cancer in mouse models using high-frequency quantitative ultrasound techniques. Ultrasound Med. Biol..

[35] Li L., Mori S., Sakamoto M., Takahashi S., Kodama T. (2013). Mouse model of lymph node metastasis via afferent lymphatic vessels for development of imaging modalities. PLoS ONE.

[36] Liu Q. (2011). Role of optical spectroscopy using endogenous contrasts in clinical cancer diagnosis. World J. Clin. Oncol..

[37] Zonios G., Dimou A., Bassukas I., Galaris D., Tsolakidis A., Kaxiras E. (2008). Melanin absorption spectroscopy: New method for noninvasive skin investigation and melanoma detection. J. Biomed. Opt..

[38] Bodén I., Nilsson D., Naredi P., Lindholm-Sethson B. (2008). Characterization of healthy skin using near infrared spectroscopy and skin impedance. Med. Biol. Eng. Comput..

[39] AŠAmar O.M., Bigio I.J. (2006). Spectroscopy for the Assessment of Melanomas. Reviews in Fluorescence 2006.

[40] Sigurdsson S., Philipsen P.A., Hansen L.K., Larsen J., Gniadecka M., Wulf H.C. (2004). Detection of skin cancer by classification of Raman spectra. IEEE Trans. Biomed. Eng..

[41] Ly E., Cardot-Leccia N., Ortonne J.P., Benchetrit M., Michiels J.F., Manfait M., Piot O. (2010). Histopathological characterization of primary cutaneous melanoma using infrared microimaging: A proof-of-concept study. Br. J. Dermatol..

[42] D Pallua J., Pezzei C., Schaefer G., Zelger B., Brunner A., Kloss-Brandstaetter A., Kloss F., Klocker H., Bartsch G., A Huck-Pezzei V. (2012). Advanced vibrational spectroscopic imaging of human tissue in life science. Curr. Proteom..

[43] Çetingül M.P., Herman C. (2011). The assessment of melanoma risk using the dynamic infrared imaging technique. J. Therm. Sci. Eng. Appl..

[44] Stępień E., Kamińska A., Surman M., Karbowska D., Wróbel A., Przybyło M. (2021). Fourier-Transform InfraRed (FT-IR) spectroscopy to show alterations in molecular composition of EV subpopulations from melanoma cell lines in different malignancy. Biochem. Biophys. Rep..

[45] Kyriakidou M., Anastassopoulou J., Tsakiris A., Koui M., Theophanides T. (2017). FT-IR Spectroscopy Study in Early Diagnosis of Skin Cancer. In Vivo.

[46] Shakya B.R., Teppo H.R., Rieppo L. (2021). Discrimination of melanoma cell lines with Fourier Transform Infrared (FTIR) spectroscopy. Spectrochim. Acta Part A Mol. Biomol. Spectrosc..

[47] Shakya B.R., Teppo H.R., Rieppo L. (2022). Optimization of measurement mode and sample processing for FTIR microspectroscopy in skin cancer research. Analyst.

[48] Srisongkram T., Weerapreeyakul N., Thumanu K. (2020). Evaluation of Melanoma (SK-MEL-2) Cell Growth between Three-Dimensional (3D) and Two-Dimensional (2D) Cell Cultures with Fourier Transform Infrared (FTIR) Microspectroscopy. Int. J. Mol. Sci..

[49] Khristoforova Y., Bratchenko L., Bratchenko I. (2023). Raman-Based Techniques in Medical Applications for Diagnostic Tasks: A Review. Int. J. Mol. Sci..

[50] Maziak D.E., Do M.T., Shamji F.M., Sundaresan S.R., Perkins D.G., Wong P.T. (2007). Fourier-transform infrared spectroscopic study of characteristic molecular structure in cancer cells of esophagus: An exploratory study. Cancer Detect. Prev..

[51] Patel J.K., Konda S., Perez O.A., Amini S., Elgart G., Berman B. (2008). Newer technologies/techniques and tools in the diagnosis of melanoma. Eur. J. Dermatol..

[52] Harland C., Kale S., Jackson P., Mortimer P., Bamber J. (2000). Differentiation of common benign pigmented skin lesions from melanoma by high-resolution ultrasound. Br. J. Dermatol..

[53] Teresa Pietrzak A., Dybiec E., Adamczyk M., Michalska-Jakubus M., Wawrzycki B., Lotti T., Rutkowski P., Krasowska D. (2015). High Frequency Ultrasonography of the Skin and Its Role as an Auxillary Tool in Diagnosis of Benign and Malignant Cutaneous Tumors—A Comparison of Two Clinical Cases. Acta Dermatovenerol. Croat..

[54] Machet L., Belot V., Naouri M., Boka M., Mourtada Y., Giraudeau B., Laure B., Perrinaud A., Machet M.C., Vaillant L. (2009). Preoperative measurement of thickness of cutaneous melanoma using high-resolution 20 MHz ultrasound imaging: A monocenter prospective study and systematic review of the literature. Ultrasound Med. Biol..

[55] Douplik A., Saiko G., Schelkanova I., Tuchin V., Jelínková H. (2013). 3—The response of tissue to laser light. Lasers for Medical Applications.

[56] Arimoto H., Egawa M. (2015). Imaging wavelength and light penetration depth for water content distribution measurement of skin. Ski. Res. Technol..

[57] Finlayson L., Barnard I.R.M., McMillan L., Ibbotson S.H., Brown C.T.A., Eadie E., Wood K. (2022). Depth Penetration of Light into Skin as a Function of Wavelength from 200 to 1000 nm. Photochem. Photobiol..

[58] Gananathan P., Rao A., Singaravelu G., Manickam E. (2017). Review of Laser in Nanophotonics ? A Literature Study for Cellular Mechanism. J. Cancer Biol..

[59] Francisco M.D., Chen W.F., Pan C.T., Lin M.C., Wen Z.H., Liao C.F., Shiue Y.L. (2021). Competitive Real-Time Near Infrared (NIR) Vein Finder Imaging Device to Improve Peripheral Subcutaneous Vein Selection in Venipuncture for Clinical Laboratory Testing. Micromachines.

[60] Ash C., Dubec M., Donne K., Tim B. (2017). Effect of wavelength and beam width on penetration in light-tissue interaction using computational methods. Lasers Med. Sci..

[61] Henderson T.A. (2024). Can infrared light really be doing what we claim it is doing? Infrared light penetration principles, practices, and limitations. Front. Neurol..

[62] Henderson T.A., Morries L.D. (2015). Near-infrared photonic energy penetration: Can infrared phototherapy effectively reach the human brain?. Neuropsychiatr. Dis. Treat..

[63] Murray K.P. (2022). Near Infrared Light Penetration in Human Tissue: An Analysis of Tissue Structure and Heterogeneities. Master’s Thesis.

[64] Barolet D., Christiaens F., Hamblin M.R. (2016). Infrared and skin: Friend or foe. J. Photochem. Photobiol. B Biol..

[65] Horton L., Brady J., Kincaid C.M., Torres A.E., Lim H.W. (2023). The effects of infrared radiation on the human skin. Photodermatol. Photoimmunol. Photomed..

[66] Scientific Committee on Emerging (2012). Newly identified health risks. Health Effects of Artificial Light.

[67] Stolik S., Delgado J., Pérez A., Anasagasti L. (2000). Measurement of the penetration depths of red and near infrared light in human? ex vivo? tissues. J. Photochem. Photobiol. B Biol..

[68] Tanaka Y., Tsunemi Y., Kawashima M., Nishida H. (2013). The Impact of Near-infrared in Plastic Surgery. Plast. Surg. Int. J..

[69] Michael A., Liakat S., Bors K., Gmachl C. (2013). In vivo measurement of mid-infrared light scattering from human skin. Biomed. Opt. Express.

[70] Briggs A., Briggs G., Kolosov O. (1995). Acoustic Microscopy.

[71] Briggs A., Briggs G., Kolosov O. (1996). Acoustic Microscopy.

[72] Wilhelm K.P., Elsner P., Berardesca E., Maibach H. (2007). Bioengineering of the Skin: Skin Imaging & Analysis.

[73] Griffiths P.R., De Haseth J.A. (2007). Fourier Transform Infrared Spectrometry.

[74] Karagiannis G., Alexiadis D., Damtsios A., Sergiadis G., Salpistis C. (2010). Diffuse reflectance spectroscopic mapping imaging applied to art objects materials determination from 200 up to 5000 nm. Rev. Sci. Instruments.

[75] Karagiannis G. Non destructive identification of art objects using multispectral images, spectra and acoustic microscopy. Proceedings of the 9th EMAS Conference: Electron Probe Microanalysis of Materials Today—Practical Aspects, with Emphasis on Cultural Heritage Application.

[76] Grivas I., Karagiannis G., Tsingotjidou A., Dori I., Grigoriadou I., Wesarg S., Georgoulias P. Experimental model for the study of melanoma Diagnostic approach with the combined use of acoustic microscopy and infrared spectroscopy, evaluated by histological analysis. Proceedings of the Joint HSBLAS-ESLAV-ECLAM Meeting Abstract Book.

[77] Zanyar Movasaghi S.R., ur Rehman D.I. (2008). Fourier Transform Infrared (FTIR) Spectroscopy of Biological Tissues. Appl. Spectrosc. Rev..

[78] Aksoy C., Severcan F. (2012). Role of Vibrational Spectroscopy in Stem Cell Research. Spectrosc. Int. J..

[79] Karagiannis G.T., Grivas I., Tsingotjidou A., Apostolidis G.K., Grigoriadou I., Dori I., Poulatsidou K.N., Doumas A., Wesarg S., Georgoulias P. Early detection of melanoma with the combined use of acoustic microscopy, infrared reflectance and Raman spectroscopy. Proceedings of the SPIE BiOS, International Society for Optics and Photonics.

[80] Anastassopoulou J., Boukaki E., Conti C., Ferraris P., Giorgini E., Rubini C., Sabbatini S., Theophanides T., Tosi G. (2009). Microimaging FT-IR spectroscopy on pathological breast tissues. Vib. Spectrosc..

[81] Dovbeshko G.I., Gridina N.Y., Kruglova E.B., Pashchuk O.P. (2000). FTIR spectroscopy studies of nucleic acid damage. Talanta.

[82] Slapničar G., Wang W., Luštrek M. (2024). Feasibility of Remote Blood Pressure Estimation via Narrow-band Multi-wavelength Pulse Transit Time. ACM Trans. Sen. Netw..

[83] Ruddell A., Croft A., Kelly-Spratt K., Furuya M., Kemp C.J. (2014). Tumors induce coordinate growth of artery, vein, and lymphatic vessel triads. BMC Cancer.

